# The ethics of clinical innovation in psychopharmacology: Challenging traditional bioethics

**DOI:** 10.1186/1747-5341-2-26

**Published:** 2007-11-08

**Authors:** S Nassir Ghaemi, Frederick K Goodwin

**Affiliations:** 1 Department of Psychiatry and Behavioral Sciences, Emory University School of Medicine, Atlanta, GA USA; 2 Department of Psychiatry, The George Washington University, Washington, DC USA

## Abstract

**Objective:**

To assess the scientific and ethical basis for clinical innovation in psychopharmacology.

**Methods:**

We conducted a literature review, utilizing MEDLINE search and bibliographic cross-referencing, and historical evidence regarding the discovery and development of new medications in psychiatry. Clinical innovation was defined as use of treatments in a clinical setting which have not been well-proven in a research setting.

**Results:**

Empirical data regarding the impact of clinical innovation in psychopharmacology are lacking. A conceptual and historical assessment of this topic highlights the ethical and scientific importance of clinical innovation. Ethically, it touches a borderline that, in our judgment, is not adequately framed in contemporary mainstream bioethics. Currently, research is viewed as not at all benefiting the patients who participate in it, while clinical care is viewed as being solely for the benefit of patients. Clinical innovation straddles these two worlds, uncomfortably at times. While many argue that clinical innovation should either be avoided or folded into research projects, we argue that clinical innovation is necessary for progress in psychopharmacology research, and that it can prosper best when guided by the following ethical principles: 1.) The treatment should be based on a viable hypothesis. 2.) Whenever possible, one's clinical observations should be reported so they can be evaluated by the scientific community. 3.) One should be willing to report unexpected observations of drug effects. 4.) A high standard of informed consent should be maintained. Again, this proposal goes against the standard view among bioethicists that research and clinical care are categorically opposed activities, as made clear by the either-or dichotomy of the Belmont Report on bioethics. This approach has so polarized our profession into clinicians versus researchers, that many clinicians will not apply new knowledge produced by clinical research until it eventually gets incorporated into formal treatment guidelines, while researchers have little to guide them as to what kind of new knowledge it is most important to provide.

**Summary:**

Clinical innovation brings out the ambiguities in our current ethical conceptions of research versus clinical care. Yet, historically, clinical innovation has been an important contributor to progress in psychopharmacology. We argue that clinical innovation should not be discouraged, but rather it should occur under certain ethical conditions.

"Almost everyone can and should do research...because almost everyone has a unique observational opportunity at some time in his life which he has an obligation to record....If one considers the fundamental operations or methods of research, one immediately realizes that most people do research at some time or another, except that they do not call their activity by that name. There are seven operations....In simple language they are counting, sorting, measuring, comparing, nature-study, guess testing, and reappraisal....Guess testing is of course what most people think of when the word research is mentioned; except that it is bad manners to call a guess a guess. It should be called an hypothesis. Let us make one plea. Guessing becomes merely a game unless it is done in the context of a plan for action. It is a waste of time elaborating untestable hypotheses [1]."

John Cade

## Introduction

Most clinicians, researchers, and ethicists would agree that it is important to expand medical knowledge, and thus, at a very basic level, it is ethical to engage in research, given appropriate protections for research subjects. As a corollary, one might argue that it is unethical not to do research, and indeed advancing knowledge through research is recognized as one of the ethical principles of the American Psychiatric Association [2]. In other words, if no one engaged in research, and no one tried to expand the bounds of knowledge, that too would be an unethical state of affairs. We must, as a result, constantly be aware of the need to balance the risk of being ignorant versus the risks involved in obtaining new knowledge. Too often, this debate is one-sided, focused on the risks involved in obtaining new knowledge. But there are risks on both sides of the ledger, and *not *doing research poses real risks also. Hence the importance of assessing the merits of clinical innovation, which we believe is a legitimate component of the research process, as explained below.

We define clinical innovation as use of treatments in a clinical setting which have not been well-proven in a research setting.

Clinical innovation occurs, by definition, outside of formal research protocols. There is a risk that guidelines of any kind, however well-intentioned, will impede clinical innovation unnecessarily. On the other hand, there are limits to acceptable innovation, and in some cases, one can imagine cases of innovation that would appear to be unethical.

Part of the problem is that the bioethics community has sought to cleanly and completely separate clinical practice from research. In the Belmont Report of the National Commission for the Protection of Human Subjects,[3] for instance, an attempt was made to separate "practice", where "interventions are designed solely to enhance the wellbeing of an individual patient or client and that have a reasonable expectation of success", from "research", defined as "an activity designed to test an hypothesis, permit conclusions to be drawn, and thereby to develop or contribute to generalizable knowledge." In fact, the clinician/researcher engaging in clinical innovation is not acting with solely one set of interests in mind, but two. On the one hand, the clinician/researcher wants to help the individual patient; on the other hand, the clinician/researchers wants to gain some experience or knowledge from his observation. Some in the bioethics community set up this scenario as a necessary conflict. They seem to think that a choice must be made: either the clinician must choose to seek only to make the patient better, without learning anything in the process, or the clinician must seek to learn something, without any intention at all to improve the patient's lot. As with so much in life, there are in fact multiple interests here and there is no need to insist that those interests do not overlap at all. First and foremost in any clinical encounter is the clinician's responsibility to the individual welfare of the patient. Any innovative treatment, observation, or hypothesis cannot be allowed to lead to complete lack of regard for the patient's welfare. Unfortunately, the Belmont Report and much of the mainstream bioethics literature presumes complete and unavoidable conflict of these interests: "When a clinician departs in a significant way from standard or accepted practice, the innovation does not, in and of itself, constitute research. The fact that a procedure is "experimental", in the sense of new, untested, or different, does not automatically place it in the category of research.... [but] the general rule is that if there is any element of research in an activity, that activity should undergo review for the protection of human subjects." It may be worthwhile to point out the dominant role of the legal profession in bioethics and its orientation towards the adversary process (winner vs. loser, guilty vs. innocent) which is predicated on artificial dichotomies in which confluence of interest becomes conflict of interest. Indeed, many bioethicists lack personal experience either in clinical medicine or in clinical research. An analogy would be if lawyers ceded legal ethics to philosophers with no experience in the practice of the law.

This approach leads, in our opinion, to *uncontrolled clinical innovation and overregulated formal research*. We will return to the Belmont Report later, but now we will turn to what we think is the key to realizing the importance and legitimacy of clinical innovation outside of formal research protocols. We think the ultimate rationale for clinical innovation is evidence from the history of psychopharmacology that such innovation is essential to the discovery of new knowledge. Further, since such innovation, by definition, occurs outside of formal research protocols, if we grant it legitimacy, then we will need to think about how we can provide an ethical framework to support it.

While we focus here on psychopharmacology, the same issues apply to medicine in general (consider for example how new surgical techniques evolve), and the same problems exist in the understanding of research ethics in medicine. Since research with psychiatric medications in particular is often subject to criticism, we believe there is a special a need to clarify this matter in psychiatry.

### The history of psychopharmacology

Every new class of agents in psychopharmacology has begun with clinical innovation or novel observations [4]. This has been the case with iproniazid, which was observed, in clinical settings, to have mood elevating properties in tuberculosis patients, and reserpine, which was observed to be associated with depression; these findings led to the development of monoamine oxidase inhibitors for the treatment of depression. Phenothiazines were used as anesthesia and a clinician observed that they had major tranquilizing effects, so they were subsequently tried as a treatment for psychosis and found to work [5]. When tricyclic antidepressants were being developed as potential treatments for schizophrenia (since they are chemical derivatives of phenothiazines), they were observed by an alert clinician to improve depressive symptoms in patients with schizophrenia [6]. Lithium was discovered by John Cade, who used it in a small group of selected manic patients (see below) [7]. And carbamazepine was initially extended to bipolar disorder based on innovative observation of evidence of benefit for mood in epilepsy [8]. In every case, when there has been a fundamental new departure in psychopharmacology, it always began with clinical innovation. It never began in a research protocol with well-thought out methodologies, hypotheses, outcome measures, and guidelines for ethical conduct.

This history is not unique to psychopharmacology. Antibiotics famously began in the serendipitous work of Alexander Fleming. Antihypertensives also were discovered based on unexpected observations. Clinical innovation is the fount of research discoveries for all of medicine, and psychiatry is no different.

Since advancing knowledge is recognized as an ethically justified and important activity, and clinical innovation precedes and is the source of major advances in formal clinical research (as noted above), then clinical innovation too is not only ethically justifiable, but is, indeed, ethically required.

If we accept that clinical innovation is ethically legitimate, then the question becomes, as with all research, what are the ethical guidelines within which clinical innovation can best be conducted, focusing on the proper assessment of the risk benefit ratio of research.

In other words, what is the ethical basis of clinical innovation? Perhaps two case scenarios will help clarify the subject.

### Case scenarios

Dr. X primarily treats mood disorders and is interested in new drugs because many of his patients have failed treatment with "standard" medications. Many new medications become available to practitioners after being approved by the FDA for disorders other than depression (e.g., epilepsy). Dr. X begins to give these medications to some of his patients with mood disorders, and soon most of his patients are taking various combinations of them. Dr. X never publishes his experience, which is unfortunate because initially studies of those medications for mood disorders are sparse. Those studies which do appear are quite preliminary of necessity (uncontrolled, nonrandomized, small case series). When asked, Dr. X strongly asserts his beliefs regarding the benefits of certain medications he uses and the lack of utility of others.

Here is another scenario. Dr. Y also likes to use new drugs, similarly for mood disorders although the medications are FDA indicated only for other conditions. After using these agents in 5–10 patients, she usually publishes her experience. Sometimes, she then becomes involved in obtaining funding for more rigorous studies of those medications which appear potentially useful based on her publications. In some cases, her early experience is confirmed by randomized studies (and occasionally new FDA indications), and sometimes they are not confirmed.

Neither Dr. X nor Dr. Y prepare a protocol or obtain IRB approval or a research-based informed consent for their clinical use of any of these medications.

Are these doctors practicing ethically?

Before we can answer this question, let's consult the history of psychopharmacology in search of a model for effective innovation.

### Innovation in psychopharmacology: lithium as a model

In the 1940s, John Cade hypothesized that mania and depression represented abnormalities of nitrogen metabolism [7]. He injected urine samples from psychiatric patients into guinea pigs, all of whom died. He concluded that the nitrogenous product, urea, was probably acting as a poison, and later tested uric acid solubilized as lithium urate, which led to marked calming of the pigs, without sedating them. Further tests identified lithium to be the calming agent, and Cade then proceeded, on the principle of primum non nocere, to try lithium himself before giving it to patients. His first patient improved markedly, but then experienced toxicity and died after a year. Despite improvement in other patients, Cade was quite concerned and abandoned using lithium further due to its toxicity, but reported his findings in detail. Other researchers, in the first randomized clinical trials in psychiatry, proved lithium safe and effective at nontoxic level.

Would we have lithium if Cade were working today? We think it is unlikely. If Cade worked in a hospital today, he likely would not have been able to obtain enough animal data to justify using lithium in humans. Hence, Cade's case highlights the opportunity costs of ethical restrictions on clinical innovation. The risks of not discovering drugs should not be ignored.

### Cade's philosophy of research

As noted in the introductory quote to this paper, drawn from a presidential address given to the Australian and New Zealand College of Psychiatry near the end of his life, Cade identified "guess testing", comparable to what we are calling innovation in this paper, as essential to psychopharmacological research. And the most essential aspect of innovation, according to Cade, is that there should be hypothesis testing. In other words, innovative use of medications should not be random; it should be driven by legitimate hypotheses, and therefore provable or disprovable. This will turn out to be important, as discussed below. The role of serendipity, driven by hypothesis testing, has also been emphasized by many [4]. Indeed, Pasteur's famous maxim about chance favoring the prepared mind can be interpreted as involving the combination of serendipity and hypothesis testing. If one has hypotheses, and one is actively seeking to test them, then one is more likely to come across "chance" findings that others may either not observe or not experience. The presence of hypotheses is not indiscriminate however. Some kind of sound rationale, be it pharmacological or theoretical is needed, to support clinical innovation.

It is also important to emphasize, nonetheless, that chance findings can also be noticed by an alert physician even if no prior hypothesis exists. For instance, no hypothesis of antidepressant effect preceded the observation that use of reserpine led to depression, or that imipramine used in schizophrenia improved depressive symptoms. These two innovative observations, neither initially driven by hypotheses, set the ball in motion that ultimately led to a Nobel Prize given to Julius Axelrod and his colleagues for their working on unraveling the biochemical mechanisms of these agents. All this work started with clinical observation without hypothesis. Much clinical innovation begins with the use of a drug for an indication not used before, which then leads to novel observations that lead to a new hypothesis that can then be tested.

Thus, while one major path of clinical innovation is the path described by Cade, where one possesses an hypothesis, and one is experimentally interested in testing the hypothesis, and new findings occur as a result. In the other path of clinical innovation, alert clinicians watch for unexpected effects when using a drug for legitimate purposes. Without possessing an initial hypothesis, these clinicians find something they didn't expect. This truly serendipitous finding leads to hypotheses which can then lead to further clinical innovation and eventually more organized research.

### Innovation and clinical trials

Most interested parties do not knowingly and overtly oppose innovation per se. Yet, if we assert that certain guidelines should encompass all innovation, we are in fact are taking a position against innovation, and not paying attention to the risks involved in not allowing sufficient innovation. If we insist that all clinical innovation should be regulated in some manner, then we are taking a purist position that focuses only on the risks of research, but not the risks of not doing research.

It is also striking that there is a double standard here: attempts to expand knowledge that are labeled "research" receive intense scrutiny, whereas clinical innovation receives no scrutiny at all. Yet the dividing line between "research" and clinical innovation is not clear-cut, and we believe that the best clinical work is also research in the sense of clinical innovation, and the best organized research grows out of clinical innovation [4].

It is vital to acknowledge that virtually everything that gets to clinical trials comes from early clinical innovation. Conceived in terms used by Evidence-based Medicine (EBM),[9] innovation in psychopharmacology more commonly proceeds bottom-up, rather than top-down (Table 1). Innovation proceeds usually from level V case reports, through levels III-IV naturalistic and nonrandomized studies, to levels I-II randomized studies.

**Table 1:** Innovation in psychopharmacology proceeds bottom-up more frequently than top-down in the levels of evidence

*Level I: Double-blind randomized trials*	Ia: Placebo-controlled
	Ib: Non placebo-controlled
*Level II: Open randomized trials*	
*Level III: Naturalistic studies*	IIIa: Nonrandomized, controlled studies
	IIIb: Large nonrandomized, uncontrolled studies (n > 100)
	IIIc: Medium-sized nonrandomized, uncontrolled studies (100 > n > 50)
*Level IV: Small naturalistic studies (nonrandomized, uncontrolled, (50 > n > 10)*	
*Level V: Case series (n < 10), Case report (n = 1), Expert opinion*	
Adapted by us from principles of Evidence-based Medicine [9]	
In terms of the timeline of how the discovery process proceeds, this schema is inverted. Everything begins at level V with novel observations and clinical innovation, and proceeds upwards. Putting emphasis on the utility of controlled studies should not lead to the conclusion that research simply consists of doing controlled studies.	

Frequently, clinical practice even outpaces or even corrects mistakes from randomized studies. For instance, in the classic case of SRI-induced sexual dysfunction, the early randomized clinical trials that led to FDA indications for those agents reported low rates of sexual dysfunction. However, clinical practice demonstrated much higher rates. In this case, the "less rigorous" evidence of clinical practice was more accurate than the "more rigorous" evidence of randomized clinical trials. This is often the case with side effects. For sexual dysfunction, a number of factors were involved: This side effect is the kind that needs to be actively assessed; passive patient self-report underestimates it. Since the clinical trials did not actively seek to identify sexual dysfunction, they did not detect it. Further, many side effects occur to a higher degree in patients with medical illnesses, or comorbid conditions like substance abuse, or in the young or the elderly, and these types of persons are generally excluded from clinical trials. Thus, the pure homogeneous clinical trial sample, which is highly selected to maximize drug efficacy, is not generalizable enough to the real world population in terms of side effects.

Hence clinical practice, and research conducted in the uncontrolled clinical setting, is highly generalizable and often produces more accurate data on certain issues, such as side effects, than do clinical trials.

### What is ethical innovation in psychopharmacology?

Conventional wisdom holds that ethical research 1) is scientifically sound; 2) involves informed consent; and 3) is not characterized by unacceptable risk. These standards are supposed to met through the institutional review board (IRB) process.

In the setting of clinical innovation, where the IRB process is not invoked, it appears to us that these three imperatives of ethical research can be met in an alternative manner. To be scientifically sound, ideally, innovation must be hypothesis-driven, as strongly urged by Cade. Further, a greater degree of informed consent should be documented in clinical notes by the innovating clinician, as compared to the use of standard proven agents. Also, the issue of risk needs to be carefully weighed and documented by the practicing clinician. This last issue is no different than the same clinical judgment exercised on a daily basis by psychiatrists with proven but risky treatments (e.g., clozapine for schizophrenia). This is not a medico-legal issue primarily, but reflects the need for more clearly educating patients about the *rationale *for what is being proposed than is often the case with non-innovative treatments. Further, the patient needs to accept the rationale, as opposed to going along with the better known risks and benefits of already proven treatments. All of these options must be explicitly discussed with patients. What must be avoided at all costs is a doctor with a new idea who simply imposes it on his patients without the above safeguards.

In some cases, clinical innovation can be ethical in the absence of an hypothesis also. This is frequently the case with extremely refractory patients. In such cases, a new treatment may be tried with no hypothesis other than a test to see if it works. We assume that the usual clinical safeguards are in place, with attention paid to risks and patient consent as described above. Of course this rationale does not cover indiscriminate or even silly practice, such as the use of an antibiotic for treatment refractory depression. As mentioned previously, some kind of sound rationale, biological or conceptual, is required to support innovative practice as well as to avoid indiscriminate activities. In this setting, all clinicians have an obligation to report unexpected novel observations, which, when at their most novel, are not hypothesis-based.

The unique risk in innovative psychopharmacology is that risks and benefits are little understood; this differs form proven but risky treatments like clozapine. In this setting, one needs to have a mindset that is cautious though not closed-minded.

### The cases

Let's illustrate these issues with our two scenarios. Dr X used all kinds of medications without attempting to organize, quantify, or publish his experience. Furthermore, he holds strong beliefs about the benefits and risks of medications based almost solely on his clinical experience. Dr. Y used the same medications, but reported her experience to the scientific community, and was involved in research protocols based on her pilot uncontrolled experience. What did Cade do? Cade used lithium with a research hypothesis in mind. He further tried it on animals before humans given the absence of any prior human use. He even tried it on himself before using it with other patients. He reported his experience immediately to the scientific community. One of his first patients in fact became toxic on lithium and died. Cade reported this death and curtailed his use of the medication. Other researchers were able to conduct rigorous research protocols based on Cade's early reports. Cade and Dr. Y have much in common, which we think helps us identify the ideal characteristics of ethical innovation.

Based on the above historical and conceptual considerations, ethical innovation, we submit, has the following ideal characteristics (Table 2). It involves the use of a new treatment based on a viable research hypothesis in an uncontrolled setting. The purpose of the innovator is not only to try to help his individual patient, but, at the very least, also to report his experience to the larger scientific community. Further, the innovator may himself be involved in advancing knowledge of that treatment beyond early uncontrolled clinical experience to more rigorous knowledge through research protocols (which then entail the usual protections of informed consent and IRB procedures).

**Table 2:** Proposed ethical guidelines for clinical innovation in psychopharmacology

1	Use of a treatment based on a viable hypothesis, with a plausible biological, empirical, or theoretical basis
2	Intention to report one's experience for the scrutiny and benefit of the scientific community
3	Willingness to report unexpected observations, perhaps initially noted without an a priori hypothesis.
4	Acceptance of a uniquely high standard of informed consent, not simply for medico-legal purposes, but on ethical grounds

Nonetheless, Dr. X is not necessarily unethical either. This is because the other pathway to innovation, though not ideal, is important. The second pathway involves pure chance observations in which unexpected findings occur without a priori hypotheses. Nor is it necessarily unethical to refrain from publishing one's experience; we simply mean to suggest that it is better to put one's experience in the public domain, preferably through publishing, so as to be open and transparent about one's activities, and also so as to spur others to re-examine and advance those observations.

### The absence of an empirical literature

We were unable to locate any empirical literature on the above topics based on a literature review, utilizing MEDLINE search with key words "ethics", "research ethics", "innovation", and "psychopharmacology", supplemented by bibliographic cross-referencing. That search did identify a number of studies which address some of these questions conceptually, [10-23] but no studies in which the topic has been examined empirically. It seems that the world of psychopharmacology research has spent little time examining the sources of innovation, and there has been little overlap between researchers and the larger world of clinicians, where non-research based decisions are made on a day-to-day basis. Yet, as the example of Cade highlights, there is a need for more overlap between clinical work and research, that is, clinical researchers who are active clinicians engaging in innovative patient care as well as structured research studies. And yet the funding structure of research, and the regulatory structure of IRBs and governmental bodies, discourages such innovative clinical researchers.

### The funding of innovative research

At the National Institute of Mental Health (NIMH), research funding has been divided between "intramural" and "extramural" types. Extramural research required extensive oversight into scientific utility. Intramural research did not require such oversight and was designed to encourage innovative ideas. In the terminology of Steve Brodie, an icon of Nobel-prize level psychiatric research, intramural research allowed investigators to "take a flier" on new ideas [24]. Unfortunately, now intramural research at the NIMH requires extramural-like levels of scientific oversight and justification. As a result, both inside the NIMH and outside, psychiatric research is more and more comprised of increasing pristine demonstrations of increasingly trivial points.

The NIMH has also tended to avoid funding of clinical psychopharmacology research on the grounds that a source of funds exists in private industry. Yet private industry targets its research to requirements of the Food and Drug Administration (FDA). There are only two purposes for an FDA indication. First, it makes a new medication available for prescribing physicians. And second, it gives a pharmaceutical company permission to market and advertise a medication for that indication. The FDA only reviews data submitted by pharmaceutical companies. Companies must have an economic incentive to go the FDA. Hence, at one level, FDA indications are purely the result of the economic decisions of pharmaceutical companies. Today, the tendency of third party payers to use FDA indications as a way to limit payment for treatments has incentivized drug companies to seek additional indications for their drugs, but in these cases too the presence or absence of an indication is primarily driven by economic, not scientific, motivations. Thus, there is minimal correlation between the amount of data supporting the use of a drug for a specific purpose and whether the drug is FDA approved for that use.

Also, the pharmaceutical industry does not generally fund long-term studies of medications, usually because of the limited length of expected marketing under patent and due to a lack of need for long-term data in terms of marketing benefit. Once a drug is on the market for an acute indication, clinicians tend to prescribe those agents for the very long-term. The prolonged use of antidepressants in unipolar depression is an example of this phenomenon. Studies of new agents such as SRIs do not exceed one year usually, and studies of tricyclic antidepressants have only been conducted for up to 5 years and even then in less than a hundred subjects. Yet on these meager grounds, it is perhaps not an exaggeration to say that millions of patients are taking these agents for decades or longer. This would appear to be ethically questionable, but those who promote more regulations on research are unintentionally contributing to inhibiting the kinds of studies that we need to conduct to remedy this situation. Unfortunately, the NIMH has rarely stepped in to fill this gap either, and the lack of solid long-term studies of major or minor psychiatric disorders is a serious problem in clinical psychopharmacology research.

Further, the Neuropharmacology division of the FDA has traditionally required rather conservative statistical paradigms, especially parallel rather than crossover designs, which do not provide information about the efficacy of new agents compared to each other. Pharmaceutical companies frequently will not pay for crossover studies, if the FDA will not allow their results to be included it the marketing of the drug. As the pharmaceutical industry is the most important source of funds for medical pharmacology research, it becomes more and more difficult to do research beyond the rigid criteria for registration, even with drugs that are already on the market.

Hence, clinicians do not have data based on FDA-oriented studies to guide many treatment decisions, and consequently clinicians are almost forced to innovate. Fortunately, the NIMH has recently expanded its use of services-based funding to obtain extensive clinical outcome data in psychiatric disorders (such as the Systematic Treatment Enhancement Program for Bipolar Disorder, STEP-BD). Further, private sources, such as the Stanley Foundation, are beginning to step forward to fill the gap with more flexible designs between the limited kinds and number of trials supported by the NIMH and the restrictive studies funded by the pharmaceutical industry.

### Issues that merit empirical study

Given that there is an absence of empirical data on which to draw, we would like to suggest specific questions that can be assessed with empirical methods.

1. How frequently do clinicians practice outside of FDA indications in psychiatry? What specific conditions tend to be treated in that setting? What specific medications are used?

2. If we accept a definition of ethical innovation along the lines we suggest, how often do clinicians practice within those guidelines?

3. How frequently are new treatments in psychopharmacology introduced by uncontrolled clinical innovation? How frequently are they introduced by research trial protocols before clinical use?

4. How frequently do research protocols derive from or depend upon prior clinical innovation or serendipitous observations?

### Relationship to formal clinical research

Some in the bioethics community seem to be unaware of the importance of clinical innovation. And thus, to them, perhaps, some of the arguments in this paper will not be convincing. We believe, in contrast, that much of the formal research enterprise depends on clinical innovation.

The argument of this paper is very simple: The only sensible reading of the historical record in psychopharmacology is that there is a factual link between clinical innovation and progress in the development of new treatments through formal clinical research. Clinical innovation is a legitimate activity, because it often serves a source of ideas and observations that later leads to classical research conducted in the formal manner of protocols, IRB reviews, and rigorous designs. Sometimes clinical practice can yield important knowledge beyond what can be gleaned from randomized clinical trial protocols. We have reviewed some historical examples, such as John Cade's discovery of lithium. Thus, clinical innovation is a legitimate and important activity. It seems to us that some critics of clinical innovation have little or no direct clinical experience, either in patient care or research, whereas those with significant experience in both activities tend to be more aware of the need for clinical innovation.

Does this approach conflict with federal standards, such as the *Belmont Report*, which has been identified by the National Institutes of Health Office of Human Subjects Research as the philosophical foundation for its ethical regulations [25]? As mentioned above, the Report leaves itself open to a strict interpretation when it asserts that "any element of research" requires formal review. However, the Report also establishes three fundamental ethical principles that are relevant to all research involving human subjects: Respect for Persons, Beneficence, and Justice. We do not see how the approach to clinical innovation outlined in this paper conflicts with these ethical principles. In fact, the absence of any ethical guidelines, as is currently the case, conflicts with these ethical principles. Thus, it may be that the status quo, which some professional medical ethicists seem to accept, is not in keeping with the principles underlying the Belmont Report. We seek to reinterpret such ethical principles in the setting of clinical innovation, whereas some in the bioethical community tend to focus on the formal mechanisms put in place to promote those principles. Even the NIH notes that the Report is "not a set of rules that can be applied rigidly to make determinations of whether a proposed research activity is ethically 'right' or 'wrong.' Rather, these regulations provide a framework in which investigators and others can ensure that serious efforts have been made to protect the rights and welfare of research subjects."

In sum, we suggest in this paper that research and clinical care should be viewed as a continuum, rather than as two categorically distinct activities. Research is *primarily *conducted to produce knowledge, and clinical care is *primarily *conducted to provide individualized care to a patient; but research also *secondarily *provides patient care, and clinical care *secondarily *produces knowledge. On that continuum, some kinds of research can actually provide a great deal of individual benefit to patients, and some kinds of clinical care can provide a great deal of new knowledge. We should give up the either-or dichotomy of the Belmont Report, which has so polarized our profession into clinicians versus researchers, and has produced clinicians who cannot apply the knowledge produced by our research, and researchers who do not know what kind of knowledge it is important to provide.

## Summary

The basic idea that underpins this paper, and which conflicts with those who promote formal regulations as the sole way to ethically conduct research, is that the best research is conducted by active clinicians, and that the best clinical work is conducted by active researchers. The strict wall separating pure research from pure clinical practice is at best a fiction, and at worst a dumbing down of both activities. Clinical innovation is the kind of activity that bridges this gap. Clinical innovation should be legitimized, accepted, and even encouraged within the framework of ethical guidelines, such as those we suggest, so as to avoid the alternative extremes of indiscriminate practice on the one hand and over-regulation of all research on the other.

## Competing interests

Dr. Goodwin currently receives research support from GlaxoSmithKline. He currently serves on the speakers bureaus of GlaxoSmithKline, Pfizer and Lilly, and is a consultant for GlaxoSmithKline, Pfizer, Lilly, Wyeth and AstraZeneca.

Dr Ghaemi currently receives research grants from GlaxoSmithKline and Pfizer. He currently serves on the speakers' bureaus of GlaxoSmithKline, Astra Zeneca, Pfizer, Janssen and Abbott Laboratories, and has served on the advisory boards of GSK, Janssen, Pfizer, Shire, and Abbott Laboratories. Neither he nor his family hold equity positions in pharmaceutical corporations.

## Authors' contributions

Both Drs. Ghaemi and Goodwin were responsible for the preparation and revision of this manuscript. Both authors read and approved the final manuscript.
